# *NFKB1* Promoter DNA from nt+402 to nt+99 Is Hypomethylated in Different Human Immune Cells

**DOI:** 10.1371/journal.pone.0156702

**Published:** 2016-06-01

**Authors:** Matthias Unterberg, Maxmiliane Julia Kreuzer, Simon Thomas Schäfer, Zainab Bazzi, Michael Adamzik, Katharina Rump

**Affiliations:** 1 Klinik für Anästhesiologie, Intensivmedizin und Schmerztherapie, Universitätsklinikum Knappschaftskrankenhaus Bochum-Langendreer der Ruhr-Universität Bochum, In der Schornau 23–25, 44892 Bochum, Germany; 2 Klinik für Anaesthesiologie, Klinikum der Universität Ludwig-Maximilians Universität München, München, Germany; Università di Napoli Federico II, ITALY

## Abstract

Sepsis, with a persistently high 90-day mortality of about 46%, is the third most frequent cause of death in intensive care units worldwide. Further understanding of the inflammatory signaling pathways occurring in sepsis is important for new efficient treatment options. Key regulator of the inflammatory response is the transcription factor NFκB. As we have recently shown, the -94 Ins/Del *NFKB1* promoter polymorphism influences sepsis mortality. However, a molecular explanation is still missing. Thus, promoter activity might be varying depending on the *NFKB1* genotype, explaining the genotype dependent mortality from sepsis, and one likely mechanism is the degree of promoter methylation. Therefore, we tested the hypothesis that NFκB mRNA expression is regulated by promoter methylation in human cell lines and primary immune cell cultures. First, we examined the methylation of the *NFKB1* promoter in U937, REH and HL-60 cells. In the promoter region of nt+99/+229 methylation in all analyzed cell lines was below 1%. Following incubation with bacterial cell wall components, no significant changes in the frequency of promoter methylation in U937 and REH cells were measured and the methylation frequency was under 1%. However, NFκB1 mRNA expression was two-fold increased in U937 cells after 24 h incubation with LPS. By contrast, demethylation by 5-Aza-2′-deoxycytidine incubation enhanced *NFκB1* expression significantly. In addition, we analyzed *NFKB1* promoter methylation in primary cells from healthy volunteers depending on the *NFKB1*–94 Ins/Del genotype. Methylation in the promoter region from nt+402 to nt+99 was below 1%. Genotype dependent differences occurred in neutrophil cells, where DD-genotype was significantly more methylated compared to II genotype at nt+284/+402. Besides in the promoter region from nt-227/-8 in ID-genotypes methylation of neutrophils was significantly decreased compared to lymphocytes and in II-genotypes methylation in neutrophils was significantly decreased compared to lymphocytes and monocytes. In addition, CHART-PCR showed that the hypomethylated promoter regions are highly accessible. Therefore we assume that the demethylated regions are very important for *NFKB1* promoter activity.

## Background

Sepsis is one of the most frequent causes of death in intensive care units worldwide and therefore a global problem [1]. It is characterized by the host’s systemic inflammatory response to infection, but the pathophysiological changes occurring in sepsis are not fully understood yet [2]. During sepsis the immune system is confronted with lots of extrinsic and intrinsic factors which cause changes of the basal state of responsiveness of lots of immune cells [3]. It is known that genetic variations could influence the outcome in sepsis [4]. A potential candidate for such variations is the gene encoding NFκB (Gene ID: 4790), since the NFκB-coupled pathway is known to amplify and perpetuate inflammatory mechanisms prevailing in sepsis [5]. We have already shown that the D allele of the functional *NF****K****B1* insertion/deletion (−94ins/delATTG) (rs28362491) polymorphism is associated with increased 30-day mortality in patients with severe sepsis [4,6,7]. Furthermore, NFκB expression differs between insertion and deletion allele carriers [4]. Unfortunately, the molecular mechanisms leading to differences in NFκB expression still remain unclear. It is known that epigenetic mechanisms, such as histone modifications and DNA methylation, play a role in the control of the immune system [8]. DNA methyl transferases (DNMTs) catalyze DNA methylation, silencing the particular gene expression. Therefore, a likely mechanism regulating NFκB expression in septic patients is DNA methylation. Due to the aforementioned facts, we tested the hypotheses that 1) NFκB mRNA expression is regulated by DNA methylation in immune cells, 2) *NFKB1* promoter methylation affects NFκB mRNA expression, 3) *NFKB1* promoter methylation is altered by inflammatory stimuli, and 4) the *NFΚB1* insertion/deletion (−94ins/delATTG) polymorphism is associated with NFκB promoter methylation.

## Materials and Methods

### Cell isolation from healthy donors

Neutrophil cells, lymphocytes and monocytes were isolated from 80 ml of venous blood obtained from healthy donors after ethics committee approval (Ethics Committee of the Ruhr University Bochum, Bochum, Germany, Reg.-No.: 4899–14) and written informed consent. In brief, cells were separated using Polymorphprep density gradient centrifugation (Progen Biotechnik, Mannheim, Germany). The upper compartment containing PBMCs and the lower compartment containing neutrophil cells were collected and washed with PBS. Monocytes were isolated using the Monocyte Isolation Kit II, human (Miltenyi, Bergisch-Gladbach, Germany). DNA from neutrophils, monocytes and lymphocytes was extracted directly. In addition Monocytes and lymphocytes were stimulated with LPS (1μg/ml) for 24 h or left unstimulated. Cells were lysed and DNA and RNA was extracted using the QIAamp DNA blood mini kit (Qiagen, Hilden, Germany) and RNeasy MiniKit (Qiagen, Hilden, Germany).

### Genotyping of healthy volunteers

Genotyping was performed as described before [6].

### Cell culture experiments with U937, HL-60, Jurkat and REH

We used the cell lines U937, Hl-60, Jurkat and REH to investigate the methylation of the *NFκB1* promoter gene in immune cells. The cells (U937, HL-60, Jurkat) were purchased from DSMZ (Leipzig, Germany) and from CLS (Eppelheim, Germany; (REH)). Firstly, we analyzed AQP5 promoter methylation after incubation with the DNA-demethylation agent 5-Aza-2'-deoxycytidin (ADC, Sigma-Aldrich, A3656). For this purpose, cells were incubated with 50 μM 5-Aza-2′-deoxycytidine (ADC) for three days and, after culture medium change, with 10 μM ADC for an additional three days. Our previous studies indicated that a seven day incubation period is required for efficient demethylation of promoters [9]. In addition, we investigated the impact of bacterial cell wall components on *NFKB1* promoter methylation. The cells were cultured in RPMI 1640 medium, supplemented with 10% FCS, penicillin (100 U/ml) and streptomycin (100 μg/ml). Cell cultures were split twice a week to maintain a cell density of about 10^6^ cells/ml. Cells in early passages (< 25) were used for all experiments, seeded in 6-well culture dishes and stimulated with either 1 μg/ml lipopolysaccharide (LPS), 10 μg/ml lipoteichoic acid (LTA) (Sigma-Aldrich Taufkirchen, Germany) or were left unstimulated (control).

Cells were lysed either 24 h and 48 h after stimulation and DNA and RNA was extracted using the QIAamp DNA blood mini kit (Qiagen, Hilden, Germany) and RNeasy MiniKit (Qiagen, Hilden, Germany).

### Methylated and unmethylated control DNA

**Fig 1:**
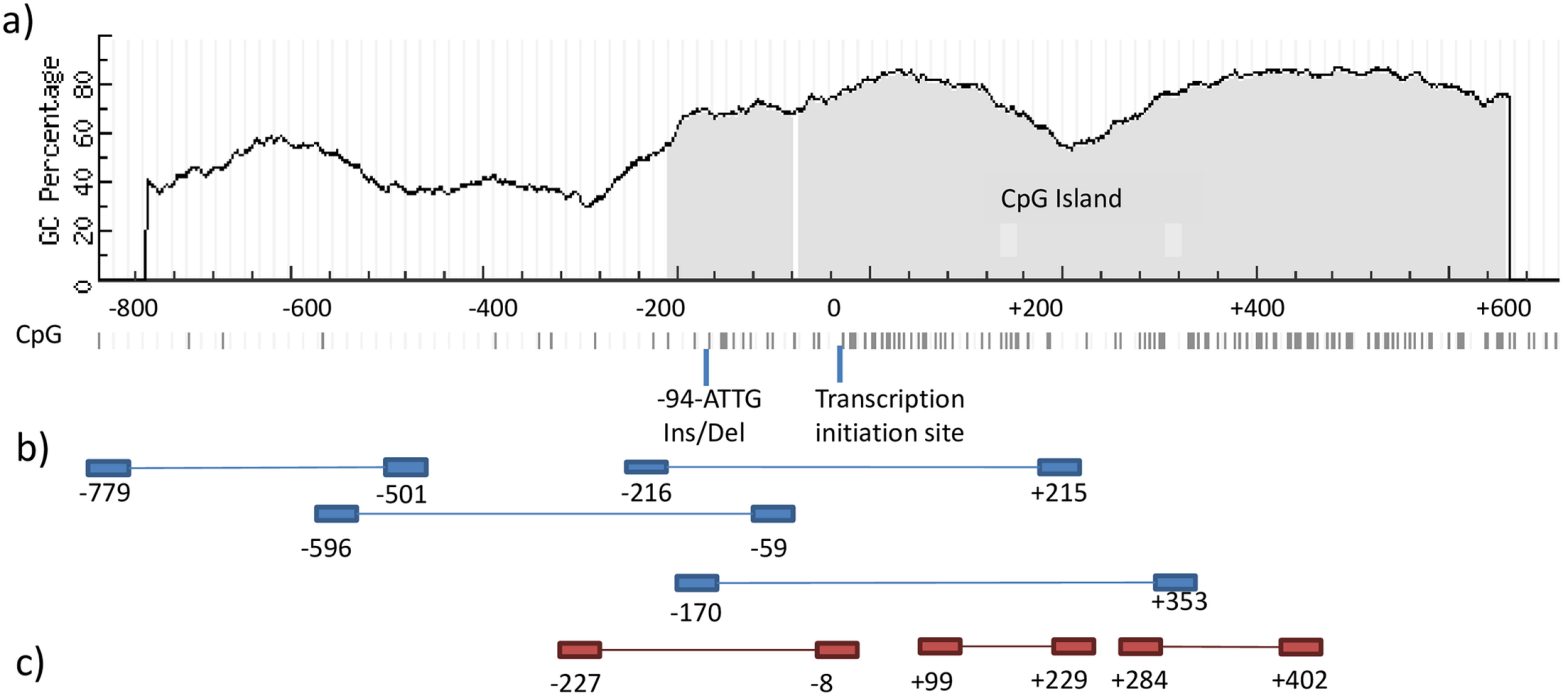
Graphic view showing primers and sequence features such as GC percent, CpG islands, and CpG site derived from MethPrimer [10] nucleotide numbers base on transcription initiation site [16] a) graphic view of the NFKB1 Promoter sequence from nt-800 to nt+600; CpG dinucleotides are indicated as bars b) graphical view of primers utilized for CHART-PCR, c) graphical view of primers utilized for methylation specific PCR.

The Cells-to-CpG^™^ Methylated & Unmethylated gDNA Control Kit (applied Biosystems, Darmstadt, Germany) was utilized to examine primer specificity. DNA was bisulfite converted with the EZ DNA Methylation-Gold^™^ Kit (Zymo research, Irvine, CA USA), following the manufacturer’s instructions. Primers (Table 1 and Fig 1) were designed using MethPrimer [10] and used for gradient PCR and products were analyzed using agarose gel electrophoresis. Methylation analysis was performed by real-time PCR [11]. The real-time PCR reaction was performed using GoTaq^®^ qPCR Master Mix (Madison, WI, USA). A DNA dilution series for methylated and unmethylated DNA was performed and a standard curve was calculated by determining DeltaCt(Ct(U)- Ct(M)) for each primer pair. According to the standard curves, the methylation was quantified using the following formulas:

$$Methylated\;DNA\;(+99/+229)\;\left[\%\right]\;=\;\frac{74,67*100}{\left(74,67+(\left(100-74,67)*EXP(-100*0,010774*DeltaCT\right))\right)}$$

$$Methylated\;DNA\;(+284/+402)\;y\;=\;\frac{92,61*100}{\left(92,61+(\left(100-92,61)*EXP(-100*0,015886*DeltaCT\right))\right)}$$

$$Methylated\;DNA\;(-227/-8)\;y\;=\;\frac{22,256*100}{\left(22,256+(\left(100-22,252)*EXP(-100*0,018369*DeltaCT\right))\right)}$$

**Table 1:** Primer pairs for CHART-PCR and Methylation-specific PCR.

Primer name	Sequence	Product size (bp)
**NFKB-779_SE**	GCC TGG TGA GGA CCT GAT TAC	278
**NFKB-501_AS**	TGATCTGGCAGAGGGGAGTT	
**NFKB -596_SE**	AGT GCC CAG CAC TAA AGC AG	537
**NFKB-59_AS**	TAG GGA AGC CCC CAG GAA G	
**NFKB-216_SE**	CTT GGA TCC ATG CCG ACC C	411
**NFKB+215_AS**	CCA CTG ACG TCG AGA GAG C	
**NFKB-170_SE**	GCTATGGACCGCATGACTCT	523
**NFKB+353_AS**	ACG GGA AGG GCA GGG GAA	
**Left M1 primer**	GTAGGAAGAGGAGGTTTCGTTATC	119
**Right M1 primer**	ACCGATAACTACGTACAAACCGA	
**Left U1 primer**	GGTAGGAAGAGGAGGTTTTGTTATT	122
**Right U1 primer**	AAACCAATAACTACATACAAACCAA	
**Left M2 primer**	TATTTGGTATGTTTGGATTTATGTC	219
**Right M2 primer**	AACTCTAACTTCCTAACAAAACGCT	
**Left U2 primer**	TTGGTATGTTTGGATTTATGTTGA	217
**Right U2 primer**	AAACTCTAACTTCCTAACAAAACACT	
**Left M3 primer**	GCGTATAGCGTTTTTAGAAGTGC	130
**Right M3 primer**	TAACTAAAAATTCCCACTAACGTCG	
**Left U3 primer**	GGGTGTATAGTGTTTTTAGAAGTGTG	130
**Right U3 primer**	ACTAAAAATTCCCACTAACATCAAA	

### Bisulfite treatment of DNA and methylation analysis

Bisulfide conversion of isolated DNA was performed with the EZ DNA Methylation-Gold^™^ Kit (Zymo research, Irvine, CA USA) following the manufacturer’s instructions. Methylation-specific PCR was performed, as described previously[11,12]. The following primer pairs were utilized to amplify methylated (M) and unmethylated (U) DNA (Table 1).

Methylation was calculated using the formulas described above.

### Real-time PCR for expression analysis

Total RNA from cells was extracted as described above and 1 μg RNA was used to synthesize cDNA. The following primers were used for *NFκB1* real-time PCR: (forward primer) 5'- CGCCGCTTAGGAGGGAGAG -3', (reverse primer) 5'- TGAAGGTATGGGCCATCTGC -3', resulting in a 204 bp fragment. Primers for the housekeeping gene actin were used as described [13]. The real-time PCR reaction was performed using GoTaq^®^ qPCR Master Mix (Madison, WI, USA). A cDNA dilution series for *NFκB* confirmed a PCR efficiency > 95%, which was comparable to the efficiency of actin. Relative *NFκB* mRNA expression was measured by two-step real-time PCR with actin as an internal control and calculated as 2^−[*Ct*(*NFκB*)−*Ct*(*β*−*Actin*)]^.

### Chromatin Accessibility Assay (CHART)

U937, REH and HL-60 cells were pelleted by centrifugation at 400 g for 5 min. Cells (5 × 10^6^ cells/sample) were washed once in ice-cold PBS. The cell pellet was resuspended in 1 ml ice-cold Nonidet P-40 lysis buffer (10 mM Tris (pH 7.4), 10 mM NaCl, 3 mM MgCl_2_, 0.5% Nonidet P-40, 0.15 mM spermine, and 0.5 mM spermidine) and incubated on ice for 10 min The suspension was centrifuged at 3000 rpm for 5 min to pellet the nuclei. The nuclei were subsequently washed in the respective digestion buffer (without CaCl_2_). DNase I, micrococcal nuclease (MNase, NEB, Frankfurt, Germany), was performed as previously described [14,15]. A control without the MNase was included for all samples. The genomic DNA was isolated using a QIAamp blood kit (Qiagen, Hilden, Germany). After DNA isolation DNA concentration was adjusted to 10 ng/μl. Subsequent PCR was carried out using OneTaq^®^ Quick-Load 2X Master Mix (NEB, Frankfurt, Germany) supplemented with 5% DMSO (Roth, Karlsruhe, Germany) using specific primers (Table 1 and Fig 1).

### *In silico* analysis

Analysis of putative transcription factor binding sites was facilitated using MathInspector (Genomatix software suite).

### Statistical analysis

Continuous parametric variables are presented as means ± standard error of the mean (SEM). The values were compared by unpaired t-test. All statistical analyses were carried out using GraphPad Prism 6 (GraphPad Software, La Jolla, USA). Differences were regarded as statistically significant with an *a priori* alpha error p of less than 0.05.

## Results

Using MethPrimer [10] one CpG island could be detected in the *NFKB*1 promoter spanning the region of nt-200 to nt+600 (Fig 1a). Primers for CHART (Fig 1b) and Methylation specific-PCR (Fig 1c) are pictured. All nucleotide numbers depend on the main transcription initiation site identified by Ten et al. [16].

**Fig 2:**
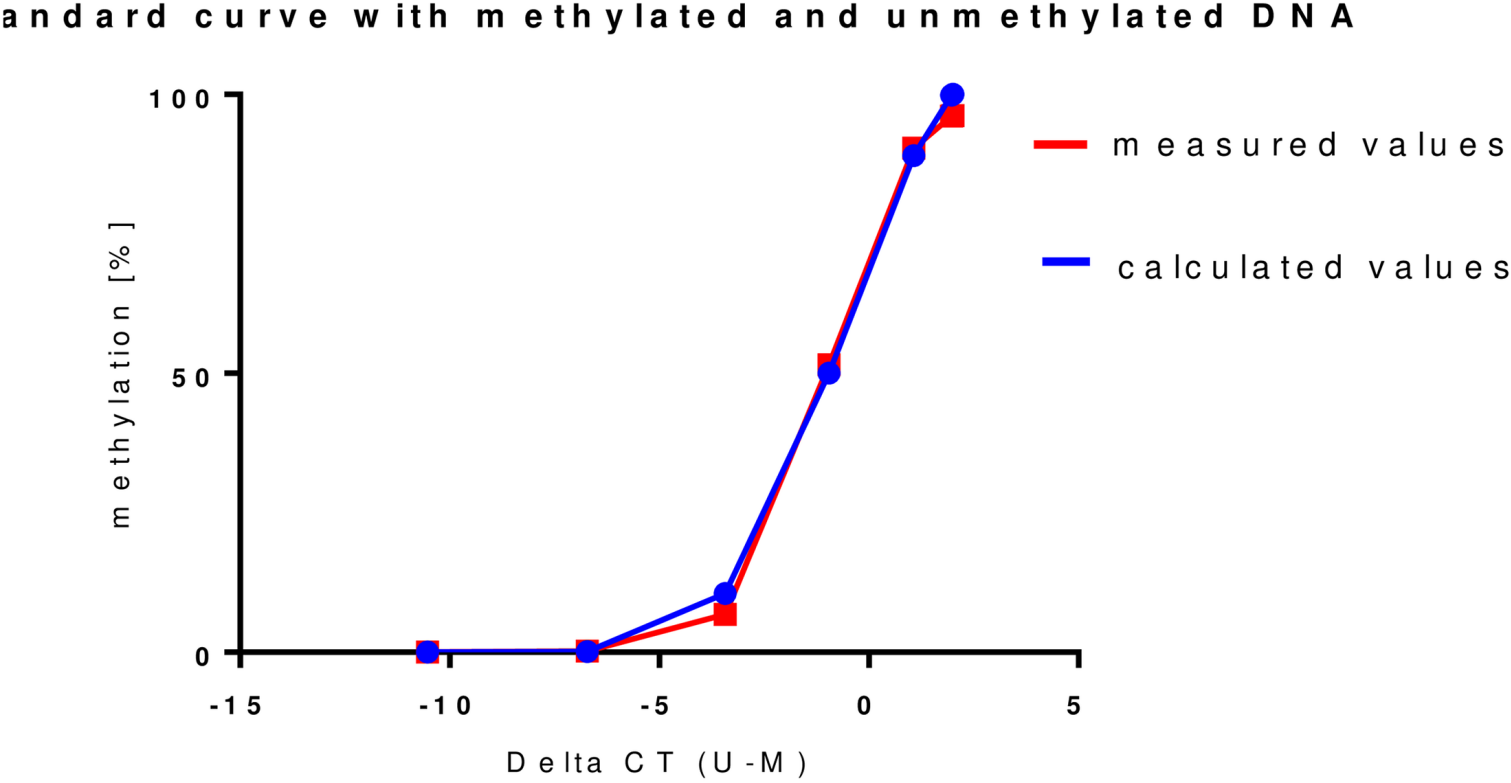
standard curve derived from the CT values measured for the nt+284/402 NFKB1 promoter region. The CT values were measured in a dilution series with defined portions of methylated and unmethylated DNA (red values). A standard curve was calculated from these values (blue values).

Three standard curves were established for quantification of the fraction of methylated molecules within the primer-binding sequences of the *NFKB1* promoter for the three different promoter regions (nt+99/229, nt+284/402, nt-227/-8). Standard curve for the region nt+284/402 is displayed (Fig 2). Reliability of reproducibility was high with a correlation coefficient r = 0.99 between two independent experiments.

**Fig 3:**
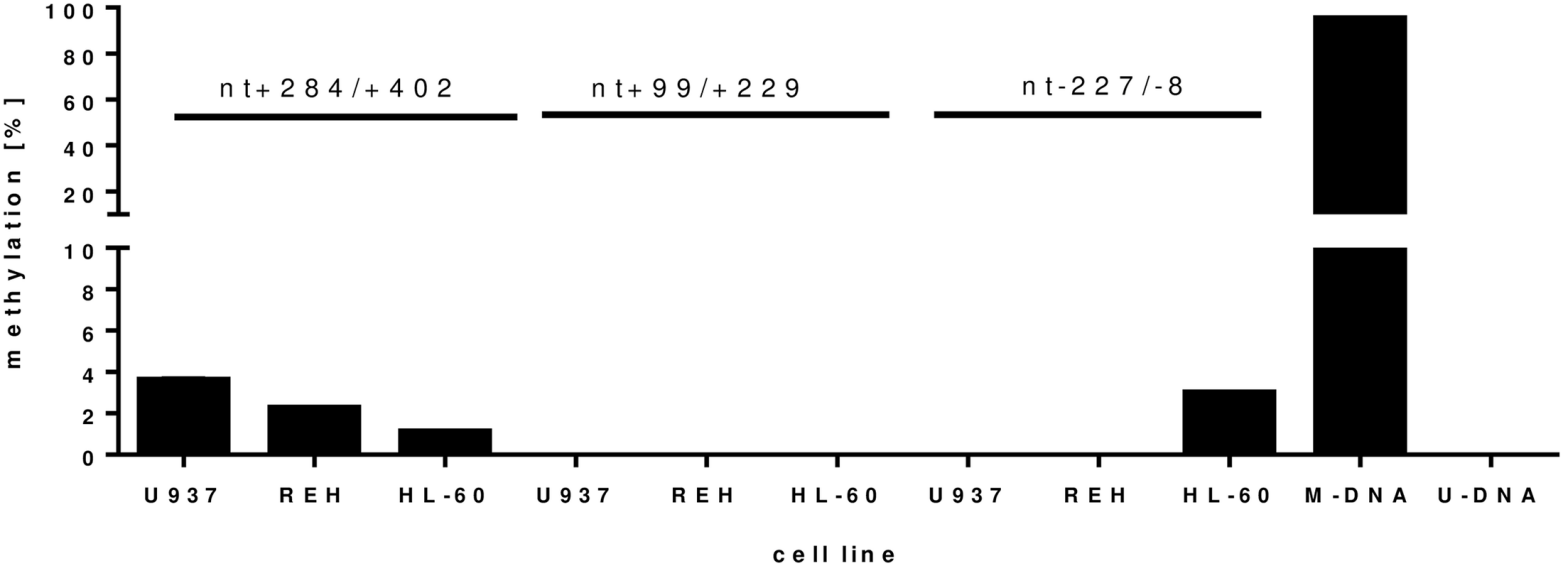
Methylation of the NFKB1 promoter in three different cell lines. Three different promoter regions were analyzed: nt+284/+402, nt+99/+229, nt-227/-8 (n = 3). A methylated (M-DNA) and unmethylated (U-DNA) was used as control.

First we analyzed the methylation of the *NFKB1* promoter in three different cell lines (REH, HL-60 and U937). The methylation of the promoter region from nt+284 to nt+402 was between 1% and 4% in the analyzed cell lines and there was no significant difference between the cell lines. In the promoter region of nt+99/+229 methylation in all analyzed cell lines was below 1%. In line with that methylation of U937 and REH cells was below 1% in the *NFKB1* promoter of nt-221/-8, where only HL-60 cells showed a methylation of 3% (Fig 3, all p>0.05).

Testing the hypothesis that NFκB1 expression is regulated by promoter methylation in immune cells, we examined the influence of inflammatory stimuli on the frequency of *NFκB1* promoter methylation in the region of nt+99/+229. The mean values of methylation in the experiments all showed low rates of NFκB1 promoter methylation ranging from 0.02 to 0.08% methylated molecules with and also without inflammatory stimulation (Table 2) in cell lines.

**Table 2:** Methylation of the +99/+229 *NFKB1* promoter region of different cell lines of human lymphocyte cells. The cells were stimulated with LPS (1 μg/ml), 10 μg/ml LTA or left unstimulated, and DNA was extracted for methylation analysis (n = 2).

	1. Measurement		2. Measurement	
	DELTA CT (U-M)	Methylation [%]	DELTA CT (U-M)	Methylation [%]
REH 24 h LPS	-11.345	0.14	-13.895	0.01
REH 24 h LTA	-12.685	0.03	-14.595	0.00
REH 24 h KO	-12.03	0.07	-12.35	0.05
U937 24 h LPS	-13.81	0.01	-12.025	0.07
U937 24 h LTA	-13.335	0.02	-11.365	0.14
U937 24 h KO	-12.365	0.05	-12.96	0.03

We isolated neutrophil cells, lymphocytes and monocytes from whole blood of twelve voluntary, healthy individuals to collateralize these results in primary human cells.

**Fig 4:**
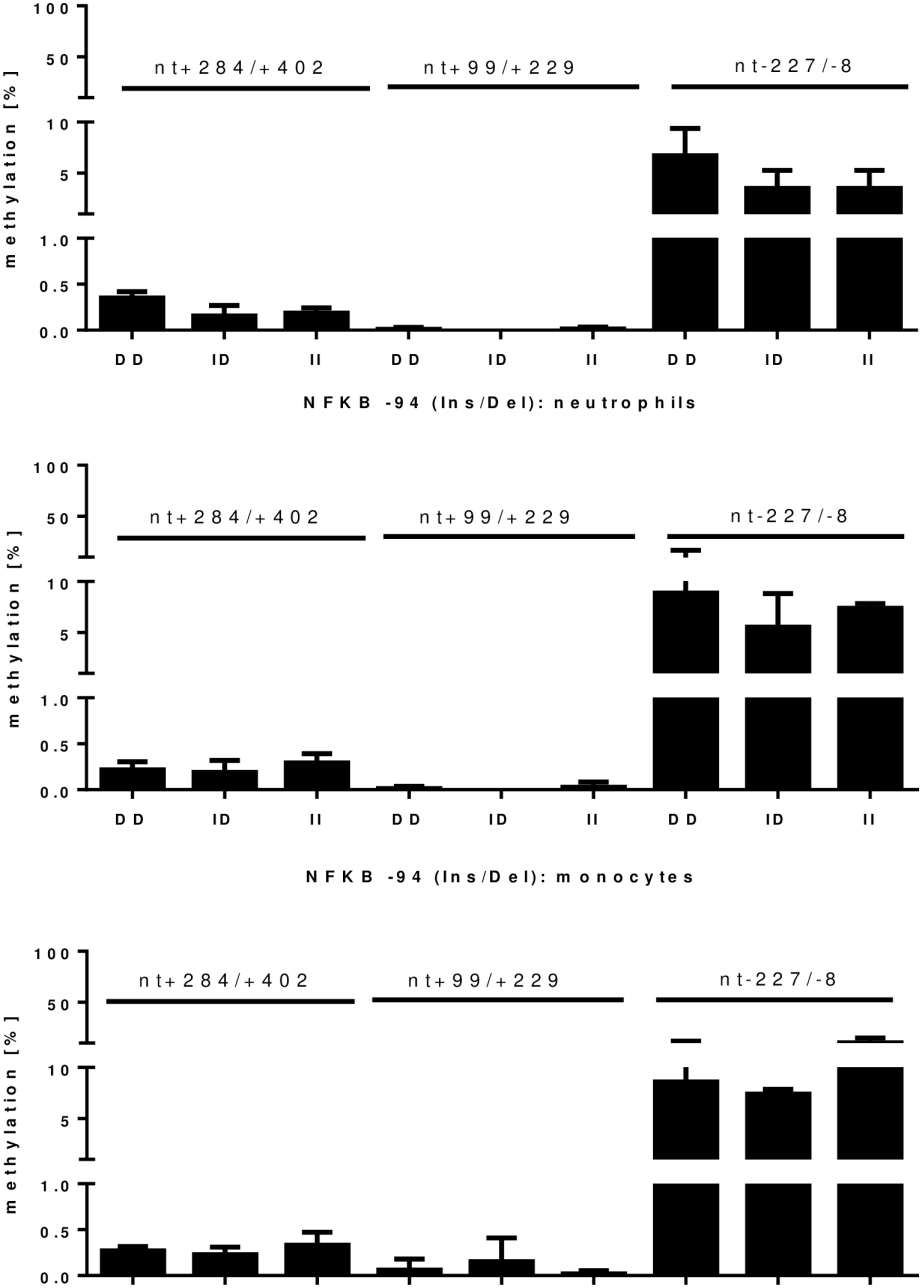
methylation of the NFKB1 promoter in different immune cells depending on the NFKB1–94 (Ins/Del) genotype (n = 12). Three different promoter regions were analyzed. NFKB1 promoter was unmethylated (<1%) from nt+402/+99. From nt-227 to -8 the methylation was about 5% to 10%.

Analysis of the methylation status dependent on *NFKB1*–94 (Ins/Del) genotype was performed in three different cell types and for three different *NFKB1* promoter regions. Methylation of the promoter region from nt+402 to +99 was below 1% in all analyzed cells. In contrast methylation from nt-227 to -8 was between 5% and 10% in the analyzed cells (Fig 4). Genotype dependent differences occurred in neutrophil cells, where DD-genotype was significantly more methylated compared to II genotype at nt+284/+402 (p = 0.0094). In addition in the promoter region surrounding the NFKB1 polymorphism from nt-227 to -8 no difference in methylation between cells was detected in DD-genotypes, whereas in ID-genotypes methylation of neutrophils was significantly decreased compared to lymphocytes (p = 0.0151) and in II-genotypes methylation in neutrophils was significantly decreased compared to lymphocytes (p = 0.0362) and monocytes (p = 0.0053) (Fig 4).

In addition *NFKB1* promoter (nt+99/+229) was examined after LPS of LTA incubation. Neutrophils, lymphocytes and monocytes of the three *NFΚB1* insertion/deletion (−94ins/delATTG) polymorphism genotypes did not show any significant differences in the methylation density either with or without inflammatory stimulation (p >0.05). The range of methylated molecules ranged from 0.05 to 0.11% in monocytes without stimulation, from 0.05 to 0.11% in neutrophils without stimulation and from 0.07 to 0.13% in lymphocytes without stimulation (all p>0.05). Thus, there was no significant difference between the genotypes. After 24 h of LPS-stimulation, monocytes (p = 0.51) and lymphocytes (p = 0.34) did not change the fraction of methylated promoter molecules significantly. Even after 48 h of LPS-stimulation, there was no significant change in methylation density compared to unstimulated cells (monocytes p = 0.39; lymphocytes p = 0.44, Table 3).

**Table 3:** Methylation of the +99/+229 NFKB1 promoter region of different blood cells isolated from healthy volunteers. Cells were isolated from fresh EDTA blood. They were then stimulated with LPS (1 μg/ml) or left unstimulated and DNA was extracted for methylation analysis (n = 3).

Methylation [%]	Proband 1	Proband 2	Proband 3
	(ID)	(II)	(DD)
**Monocytes**	0.05	0.11	0.06
**Monocytes after 24 h LPS**	0.06	0.06	0.06
**Monocyts after 48 h LPS**	0.03	0.21	
**Neutrophils**	0.09	0.05	0.11
**Lymphocytes**	0.07	0.07	0.13
**Lymphocytes after 24 h LPS**	0.06	0.04	0.09
**Lymphocytes after 48 h LPS**	0.09	0.10	

**Fig 5:**
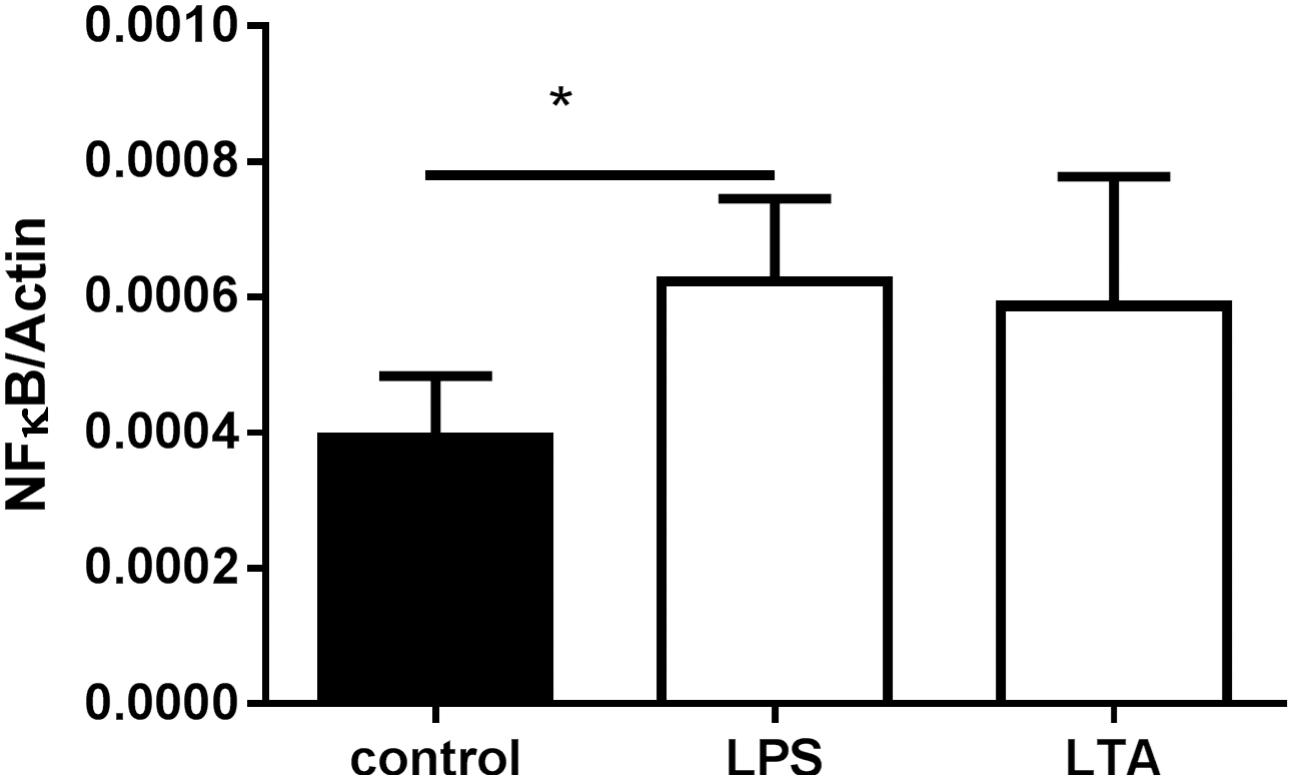
NFκB expression in U937 cells 24 h after stimulation with 1 μg/ml LPS or 10 μg/ml LTA (n = 4).

Both Gram-negative (LPS) and -positive (LTA) stimulation showed an impact on the *NFκB1* expression. The NFκB1/β-actin ratio in U937 cells showed a two-fold increase in *NFκB1* expression following the 24 h incubation with Gram-negative or -positive substance (Fig 5, p = 0.047 unstimulated vs LPS-stimulated; p = 0.1 unstimulated vs LTA-stimulated).

**Fig 6:**
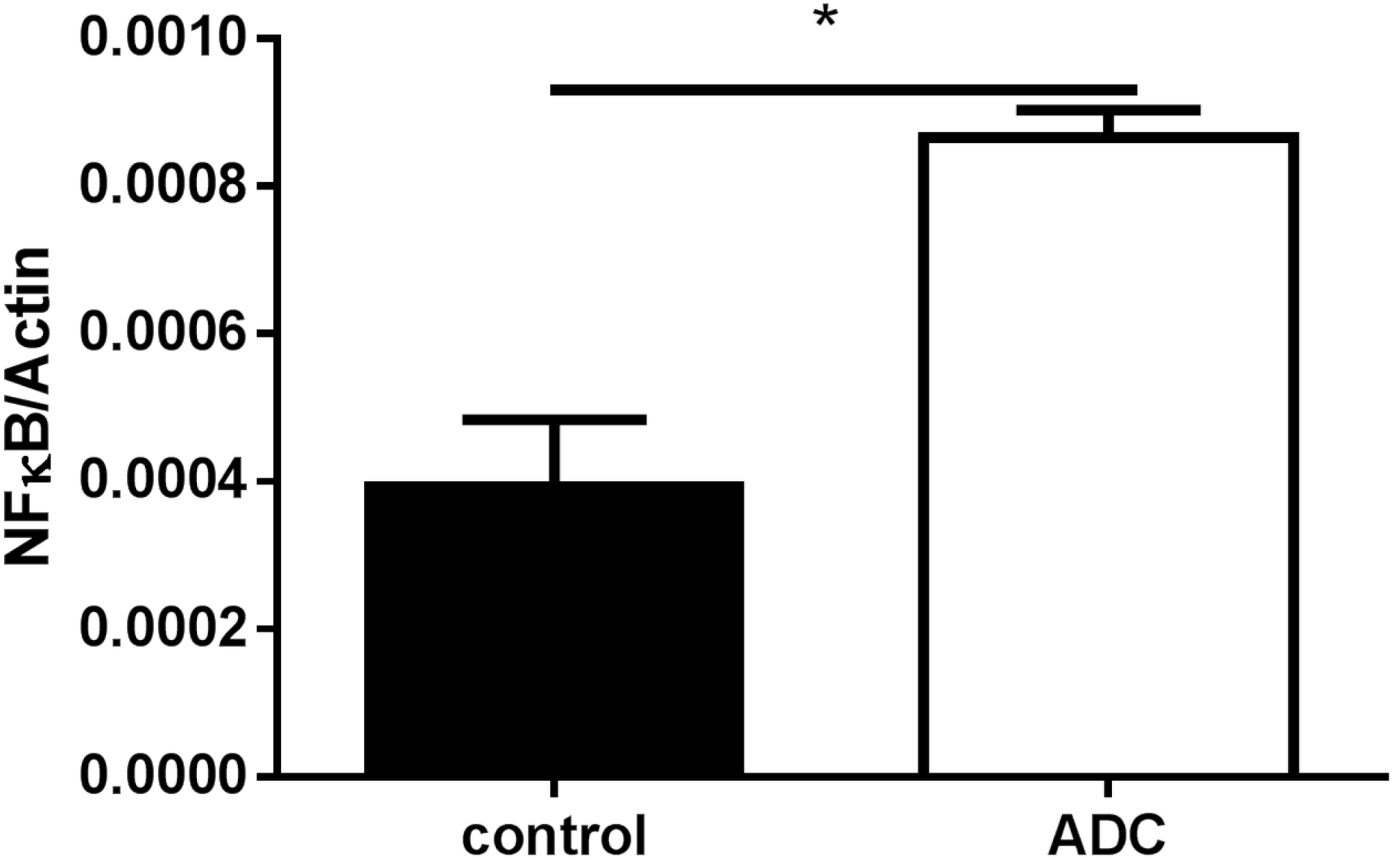
NFκB expression in U937 cells. Cells were left unstimulated (U937) or incubated with a final concentration of 10 μM ADC for seven days. (n = 4).

Finding no significant changes in the methylation status of NFκB1 promoter in cell lines after LPS, we tested whether the insertion of 5-desoxy-aza-cytidine (ADC), which replaces cytidine in the genomic DNA and cannot be methylated by DNMTs, has the potential to increase the *NFκB1* expression. After seven days, we detected a two-fold increase in the ratio of *NFκB1/ß-actin* expression after ADC incubation in U937 cells (Fig 6, mean of three independent experiments, p = 0.0069). Thus the demethylation of the cellular DNA influences the *NFκB1* mRNA expression.

**Fig 7:**
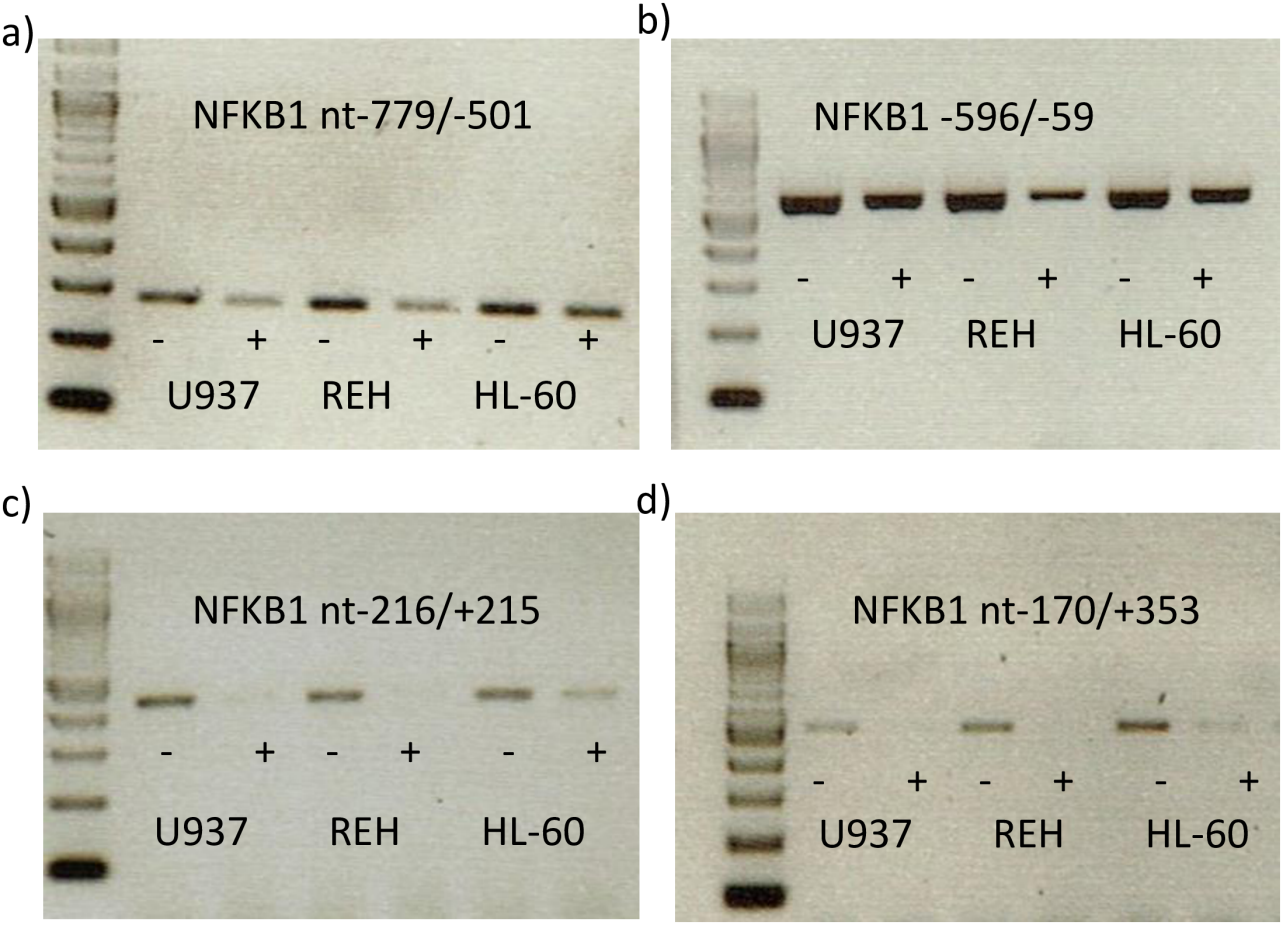
chromatin accessibility (CHART) of different regions of the NFKB1 promoter. Chromatin from different cell lines was incubated with MNase (+) or left untreated (-) and DNA was extracted. PCR of different NFKB1 promoter regions as performed.

To conclude we analyzed the accessibility of the *NFKB1* promoter by CHART-PCR. Promoter region of nt+779/+501 was mainly inaccessible for MNase digestion as shown by PCR (Fig 7a) in all analyzed cell lines. In REH and U937 cells DNA became accessible from nt-216 (Fig 7b, 7c and 7d) and DNA from HL-60 cells was accessible from nt-170 (Fig 7d).

## Discussion

In this study, we analyzed the *NFKB1–94(Ins/Del)* genotype dependent promoter methylation. Thus, we are the first to show, that *NFKB1* promoter methylation differs in II and DD genotypes in neutrophil cells. Therefore genotype dependent differences in patients outcome and the inflammatory response may result from promoter methylation in immune cells [17,18]. We further showed that the methylation of the *NFKB1* promoter methylation is reduced in neutrophil cells from II and ID genotypes compared to lymphocytes and monocytes. In contrast incubation with inflammatory stimuli of both a human lymphoid cell line (REH) and a human monocytic cell line (U937) and primary human blood cells did not show any significant differences in the methylation density of the *NFKB1* promoter. Another important result of our study is, that the *NFKB1* promoter region from nt+402/+94 is hypomethylated as it was below 1% in primary samples. In addition this promoter region was shown to be highly accessible as it was shown with CHART-PCR. In line with that the promoter region containing the polymorphism was highly accessible and methylation was below 10% in the analyzed samples. Therefore these regions might play an important role in NFκB expression.

The hypomethylation in the *NFKB1* promoter might be reasonable because *NFκB1* is consecutively expressed in immune cells. NFκB1 is present in cells in an inactive state and do not require new protein synthesis in order to become activated. Furthermore, the NFκB1 is not only involved in inflammatory processes, but also in several other cellular signaling pathways [19–21]. Therefore, downregulation of *NFκB1* mRNA expression by methylation might impact on cellular pathways, which regulate expression of genes involved in embryo or cell lineage development or cell cycle progression, which are required for survival.

In contrast to our hypothesis that NFκB is not regulated by *NFΚB1* promoter methylation, we found that demethylation of DNA with the demethylation agent ADC let to an increased amount of NFκB expression. Therefore we can assume that demethylation with ADC might further demethylate the *NFKB1* promoter region from nt-227/-8, which might further increase *NFKB1* promoter activity. Additionally, this might be due to upregulation of several other factors, which are affected by demethylation as well.

Thus it need to be questioned whether other genes involved in the LPS signaling pathway in immune cells are regulated by methylation. Studying the current literature, we found that some transcription factors, which are induced after LPS incubation in immune cells [22] are indeed regulated by methylation. As an example, SRF (serum response factor) and Elk1 (member of the ETS oncogene family) are regulated by promoter methylation (Table 4 [23]), while other genes, such as the transcription factor CREB (cAMP response element binding protein), are not regulated by methylation of its promoter (Table 4). In addition, there are numerous hints that important inflammatory genes, such as IL-6, IL-8 Tnf-α and the Toll-like receptors (TLRs) 2 and 4, are regulated by methylation of their promoters (Table 4 [24–27]).

**Table 4:** Overview of literature of regulation by methylation of important transcription factors and inflammatory genes.

Gene/protein name	Regulated by methylation	Reference
**ELK1**	Yes, downregulated in cancer	[23]
**SRF**	Yes, downregulated in cancer	[23]
**CREB**	To the best of our knowledge, there are no data available	
**IL-6**	Promoter demethylation after ADC, LPS has no additional effect on demethylation, one position: nt-1099 is less methylated in PBMCs of patients with rheumatoid arthritis	[29,30]
**IL-8**	7.0-fold increased expression after LPS with ADC-TSA (trichostatin A) treatment	[24]
**IL-6**	2.5-fold increased expression after LPS with ADC-TSA (trichostatin A) treatment	[24]
**TNF-α**	1.6-fold increased expression after LPS with ADC-TSA (trichostatin A) treatment	[24]
**TLR-2**	Hypomethylation is associated with increased proinflammatory response	[25]
**TLR-4**	TLR-4 hypermethylation causes low response to LPS	[26]
**TLR-2, TLR-4, lipopolysaccharide-binding protein (LBP) and haptoglobin (HP)**	Promoters are opened after LPS, which may be caused by demethylation	[27]

In contrast to these findings in literature, *NFκB1* demethylation was not responsible for increased activation of *NFκB1* after an inflammatory stimulus such as LPS [4,22]. In this study, we now could demonstrate that LPS did not alter *NFKB1* promoter methylation. This is not surprising, because as mentioned above NFκB1 is present in cells in an inactive state and do not require new protein synthesis in order to become activated. The rapid activation of *NFκB1* after LPS is facilitated by the nuclear translocation of NFκB dimers after degradation of the inactivator IKKB [22].

We can only speculate about the meaning of the lack of *NFκB1* methylation, which is not altered after LPS. Analyzing the hypomethylated *NFκB1* promoter sequence, we found several putative binding sites for SP-1, CREB and NFκB1 in this promoter region. Therefore, rapid activation and ubiquitary expression of NFκB1 might be facilitated by hypomethylation and binding of these factors.

In summary, we show for the first time that the *NFκB1* promoter is hypomethylated and that methylation of neutrophils depends on NFKB1–94 (Ins/Del) genotype. This might be important for novel therapeutic approaches which use demethylation of other genes. A novel study could show that aberrant DNA methylation in lung tissue samples from rats is associated with altered outcome of acute respiratory distress syndrome [28]. Therefore, it might be beneficial for therapies with DNA-methylation-altering pharmaceuticals that the NFκB signaling pathway is not altered by this therapeutic.
